# New Appliances and Things Medical

**Published:** 1898-05-28

**Authors:** 


### NEW APPLIANCES AND THINGS MEDICAL.

[We shall be glad to receive, at our Office, 28 & 29, Southampton Street, Strand, London, W.C., from the manufacturers, specimens of all new preparations and appliances which may be brought out from time to time.]

### EMALIN, A NEW FERRUGINOUS PREPARATION.

(William Poppelreuter, 19, St. Dunstan's Hill, E.C.)

A little time since we mentioned in these columns a new vaseline preparation called Valsol manufactured by the same firm. The success which has followed its more extended use fully justifies what we then said about it, and encourages one to take a favourable view of the companion speciality of the same firm. Emalin (Dr. Dahmen), better known on the Continent as "Hæmalbuminum," is practically digested blood. It is soluble, may be regarded as a highly concentrated food, and has therapeutic properties which have been proved to be of special value in chlorosis, phthisis, anæmia, &c. The average dose for an adult is 15-30 grains daily. It may be taken as a dry powder, as a lozenge, in enemata, or mixed with wine. In all cases where iron is indicated, but especially in cases where there is anæmia combined with indigestion, Emalin will be found of the greatest use as a hæmatic tonic and food.

### NURSE SLATTER'S IMPROVED SANITARY BED-PAN.

(The Nurse Slatter Company, Hermitage House, Bath Road, Cheltenham.)

This is a bed-pan provided with an inflatable india-rubber air cushion, by which all the discomforts caused by the pressure of the bed-pan against the back are done away with. The cushion can be easily removed for cleansing purposes. Owing to the elasticity of the cushion the lid fits quite tightly and prevents all escape of odour. The outlet is of large siz', and the plug is in the form of a cup, which may be made to contain a supply of disinfectant to pour over the con'ents of the pan immediately after use. The air cushion is furnished with a valve by which it can be inflated afresh if it should get too soft, much after the manner of a bicycle tyre. We are afraid that the pan will be found rather thick, and that the cleaning of the india-rubber will be somewhat troublesome, but we have no doubt whatever that the arrangement will be found very comfortable by the patient, which after all is the great point, and will be especially useful in those paralytic cases in which the nutrition of the skin is so interfered with that bed sores are peculiarly liable to be produced.

### MALT EXTRACT.

(Distillers' Company, Limited, Edinburgh.)

We have received a further sample of the above malt extract. In January of last year we gave an analysis of the same in these columns; the sample we have before us appears in all respects to come up to the same standard of excellence. It constitutes a pleasant digestive adjuvant and food, and is to be specially recommended for children and those in whom the general nutrition has suffered from wasting diseases, or excessive bodily or mental strain.

### PRESERVED CREAM.

(Dahl Milk Company, Drammen, Norway.)

We have received a sample tin of the above brand of double thick cream. We find it of excellent quality, and for practical purposes undeteriorated by the processes to which it has been submitted for the purposes of preservation. When removed from the tin and briskly whisked it will be found undistinguishable from ordinary fresh cream. If kept in a cool place, it is guaranteed to keep sweet for six months. For tea, coffee, cooking, and other purposes we recommend this useful innova'ion to the notice of our readers.

